# Key bacterial families (Clostridiaceae, Erysipelotrichaceae and Bacteroidaceae) are related to the digestion of protein and energy in dogs

**DOI:** 10.7717/peerj.3019

**Published:** 2017-03-02

**Authors:** Emma N. Bermingham, Paul Maclean, David G. Thomas, Nicholas J. Cave, Wayne Young

**Affiliations:** 1 AgResearch Ltd., Food Nutrition & Health Team, Palmerston North, New Zealand; 2 AgResearch Ltd., Bioinformatics & Statistics Team, Lincoln, New Zealand; 3 Massey University, Institute of Veterinary, Animal & Biomedical Sciences, Palmerston North, New Zealand

**Keywords:** Dog, Faecal microbiota, Nutrient digestibility

## Abstract

**Background:**

Much of the recent research in companion animal nutrition has focussed on understanding the role of diet on faecal microbiota composition. To date, diet-induced changes in faecal microbiota observed in humans and rodents have been extrapolated to pets in spite of their very different dietary and metabolic requirements. This lack of direct evidence means that the mechanisms by which microbiota influences health in dogs are poorly understood. We hypothesised that changes in faecal microbiota correlate with physiological parameters including apparent macronutrient digestibility.

**Methods:**

Fifteen adult dogs were assigned to two diet groups, exclusively fed either a premium kibbled diet (kibble; *K*; *n* = 8) or a raw red meat diet (meat; *M*; *n* = 7) for nine weeks. Apparent digestibility of macronutrients (protein, fat, gross energy and dry matter), faecal weight, faecal health scores, faecal VFA concentrations and faecal microbial composition were determined. Datasets were integrated using mixOmics in R.

**Results:**

Faecal weight and VFA levels were lower and the apparent digestibility of protein and energy were higher in dogs on the meat diet. Diet significantly affected 27 microbial families and 53 genera in the faeces. In particular, the abundances of *Bacteriodes*, *Prevotella*, *Peptostreptococcus* and *Faecalibacterium* were lower in dogs fed the meat diet, whereas *Fusobacterium*, *Lactobacillus* and *Clostridium* were all more abundant.

**Discussion:**

Our results show clear associations of specific microbial taxa with diet composition. For example, Clostridiaceae, Erysipelotrichaceae and Bacteroidaceae were highly correlated to parameters such as protein and fat digestibility in the dog. By understanding the relationship between faecal microbiota and physiological parameters we will gain better insights into the effects of diet on the nutrition of our pets.

## Introduction

Pet dogs face the same nutrition-related maladies as humans (e.g., obesity and diabetes (German, 2006)), and are living longer. World-wide, pet dogs are increasingly considered as family members, and consequently there is increasing focus on the health benefits of their diets. Dogs have no nutritional requirement for carbohydrates (Association of American Feed Control Officials, 2016) and have evolved to thrive on a diet high in animal protein and fat (Buffington, 2008; Verbrugghe et al., 2012). However, dogs can tolerate low levels of carbohydrates and are therefore classed as omnivorous carnivores (Swanson et al., 2011). The canine intestinal tract reflects its carnivorous evolutionary history, being relatively short and thick-walled (Bosch, Hagen-Plantinga & Hendriks, 2015). Similarly, the composition of dogs’ intestinal microbiota may reflect these unique dietary requirements.

In humans and rodents, there is a clear association between microbiota and diseases such as diabetes and obesity (Hartstra et al., 2015; He, Shan & Song, 2015; Duran-Pinedo & Frias-Lopez, 2015). Consistent with this, over the last five years much of the research in companion animal nutrition has focussed on understanding the role of diet on the composition of the faecal microbiota (Deng & Swanson, 2015). To date, diet-induced changes in faecal microbiota observed in humans and rodents have been extrapolated to dogs and cats, in spite of their very different dietary requirements. This means there is a lack of species-specific data on how microbiota influence health in carnivorous pets. Recent global metagenomic screening studies have shown that carnivores, such as dogs, have different metabolic pathways associated with butyrate production (Vital et al., 2015). This suggests that interpretation of the effect of diet on intestinal health may require a species-specific approach.

On this basis, the aim of this study was to integrate diet-associated changes in microbial composition with phenotypic data such as apparent nutrient digestibility and faecal health score. This approach may help understand how microbial and host physiology intersect, thereby increasing the understanding of dietary effects on microbial composition in the dog, and their impact on nutrition (Deng & Swanson, 2015).

## Materials and Methods

### Animals and diets

**Figure 1:**
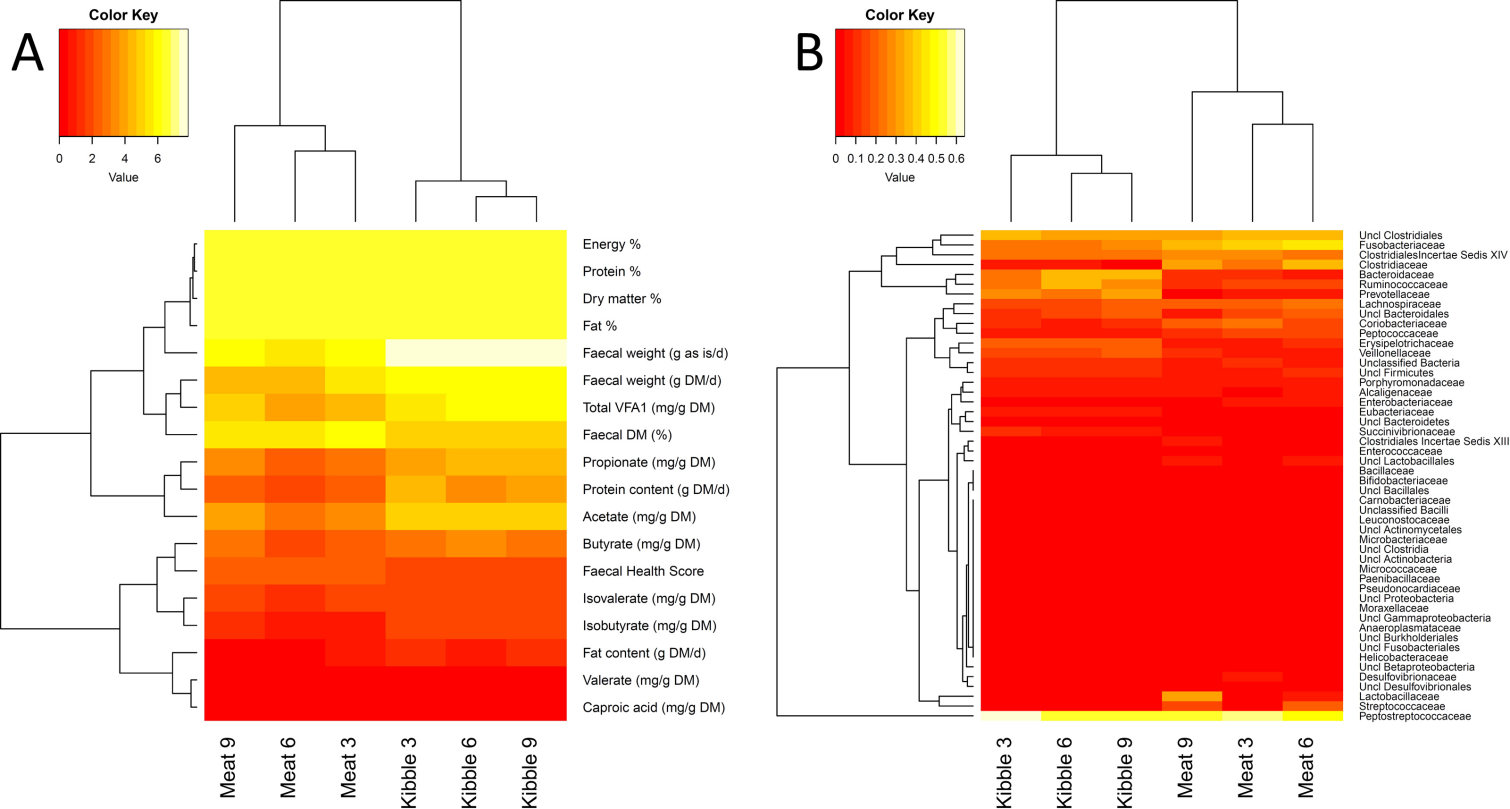
Heat maps indicating study design. Heat maps faecal bacteria levels in the dog (reported at the family level) and physiological markers of intestinal function in dogs fed both the kibbled and meat diet. To enhance the display of a large range of values in both cases, the log_2_ transformed macronutrient data and square root of the bacterial family data are displayed.

The protocol for this study was approved by the Massey University Animal Ethics Committee (MUAEC 14/37). Sixteen mature, healthy dogs (*Canis familiaris;* Harrier Hounds; mean age: 5.81 years, SE: 0.72; 21.8–31.9 kg) were randomly selected from the colony at the Centre for Canine Nutrition at Massey University, Palmerston North. Dogs were housed in groups (*n* = 4) in large outdoor pens (10 m × 10 m) during the day, and in pairs in indoor pens (2 m × 2 m) with access to an attached outside area (2 m × 2 m) at night. One dog was removed from the study for reasons unrelated to the study. The study design is outlined in Fig. 1 and Fig. S1.

The dogs were fed one of two adult maintenance diets (Table 1) throughout the trial period: a commercially available kibbled diet (Kibble, 3 males and 5 females) or a raw red meat diet (73% beef muscle, 10% beef liver, 5% bone chip, 5% beef tripe, 3.5% beef heart, 3.5% beef kidney, 0.2% mineral pre-mix; Meat, 2 males and 5 females). Both diets were formulated to meet the nutrient requirements for maintenance according to the Association of American Feed Control Officials (2016). Daily dietary energy intake offered to each dog was initially calculated as ME (kJ) = 552× kg BW^0.75^ (Association of American Feed Control Officials, 2016), and was adjusted weekly to maintain a consistent body weight. Water was available *ad libitum* at all times.

**Table 1:** Macronutrient profiles of diets. Macronutrient profiles of a commercially available kibbled and red meat diet fed to the domestic dog (*Canis familiaris*) for nine weeks.

Nutrient	Kibble	Meat^a^
Dry matter (DM) %	91.5	24.7
Crude protein % DM	29.9	76.3
Crude fat % DM	27.1	17.9
Crude fibre % DM	2.4	0.6
Crude ash % DM	6.0	4.6
NFE^b^ % DM	34.6	0.6

**Notes.**

a 73% beef muscle, 10% beef liver, 5% bone chip, 5% beef tripe, 3.5% beef heart, 3.5% beef kidney, 0.2% mineral pre-mix.

b Nitrogen free extract calculated by difference (100—crude protein—crude fat—crude fibre—ash).

To determine the apparent digestibility of energy, dry matter, carbohydrate, protein and fat, individual food intake and refusals were recorded daily and total faecal output was collected over a four day period on weeks 3, 6 and 9 (Association of American Feed Control Officials, 2016). For each digestibility period, a sub-sample of each diet and the faeces collected were frozen (−20 °C), freeze-dried and ground before macronutrient analysis. After the digestibility period, a fresh faecal sample was collected from each dog within 10 min of excretion and stored at −85 °C to measure volatile fatty acids (VFA) and faecal microbial composition.

### Laboratory analyses

#### Macronutrient analysis

Diets and faecal samples were analysed for percentage moisture using a convection oven at 105 °C (AOAC 930.15, 925.10), and percentage ash residue was determined using a furnace at 550 °C (AOAC 942.05). Crude protein and crude fat were determined using the Leco total combustion method (AOAC 968.06), and acid hydrolysis/Mojonnier extraction (AOAC 954.02) respectively. Gross energy (kJ/g) was determined using bomb calorimetry. Crude fibre was determined using the gravimetric method (AOAC 978.10) and nitrogen-free extractable matter (NFE) by difference (Deng & Swanson, 2015). Dry matter (DM) was calculated as 100 less the % moisture. The apparent digestibility of energy, protein, fat and dry matter was calculated as described previously (Bermingham et al., 2013b).

#### Faecal VFA

Faecal VFA (acetate, propionate, butyrate, isobutyrate, isovalerate and valerate) were determined as described previously (Wronkowska et al., 2006). Briefly, the samples were de-proteinised using metaphosphoric acid. The supernatant was injected directly into gas chromatography (Shimadzu GC17-A, capillary column Alltech ATTM-1000, 15 m × 0.53 mm ID, 1.00 µm film) with hydrogen as the carrier gas, FID detector, and iso-caproic acid as an internal standard.

### Faecal microbial composition

DNA was extracted from faecal samples using the NucleoSpin Soil kit following the manufacturer’s instructions (Macherey Nagel, Düren, Germany). Faecal microbiota profiles were determined by pyrotag sequencing of the V4–V6 region of the bacterial 16S rRNA gene using a previously described method (Young et al., 2015).

### Statistical analysis and dataset integration

Food intake, body weight, apparent nutrient digestibility, faecal health score, faecal weight and faecal VFA concentrations were analysed using repeated measures Correlation Models by REML using treatment (meat (*M*) vs kibble (*K*)) and time (week of study) as factors (Genstat version 17.1).

Faecal microbial amplicon sequences were processed using Qiime 1.8 (Caporaso et al., 2010). Reads were quality filtered using default settings and sequences were chimera checked using the USEARCH method against the Greengenes alignment (release GG_13_8). Chimeric sequences were removed from subsequent analyses. Sequences were clustered at 97% similarity into operational taxonomic units (OTUs) using the UCLUST method and representative sequences were assigned taxonomies using the RDP classifier. Differences between mean relative abundance of taxa between treatments were analysed by two-factor ANOVA (diet × time) and single factor (diet) permutation ANOVA using the RVAideMemoire R package with 2,000 permutations (Hervé, 2014).

Samples which did not have complete data for the physical measurement, metabolomics, and 16S amplicon data were removed from the integrated analyses. This resulted in a dataset with 24 samples matched by dog and time point. Variables that added no unique information to the analyses due to zero variance, notably in the diet characteristics as shown in Appendix 1, were removed. These columns correlate 100% with each other, which was not suitable for many of the integration techniques which rely on correlation.

The 16S rDNA amplicon sequencing data was reduced further by using the “nearZeroVar” function in the mixOmics R package (González et al., 2012), which removed columns with only one non-zero value and columns which had very few unique values (essentially non-zero values that that do not generate substantial variation) relative to the number of samples, because these were neither informative nor non-redundant. The mixOmics package in R was used to perform all data integration, while the principal component analyses (PCAs) were performed using standard R command (R Core Team, 2015). PCA was used to detect patterns in each single high-dimension dataset. Canonical Correlation Analysis (CCA) was used to compare two (and in cases of regularisation, where complexity is penalised, more than two) high-dimension datasets. Shrunk CCA was used for the integration of faecal 16S rDNA amplicon data and combined metabolomic and physical measurement data to account for a large amount of co-correlation between the metabolomic and physical measurement data.

We used CCA to measure the association between the two groups of variables: the relative abundances of microbial taxa and the phenotypic data (which include metabolites, dietary composition data, and faecal characteristics). CCA attempts to extract associations between the two sets of variables, over and above the simple pairwise correlations, from this correlation matrix. In other words, CCA finds the correlation(s) between the dataset of original variables adjusting for the within-dataset correlations, and these canonical correlations can be thought of as ‘layers’ of correlations or a number of possible ‘links’ between the two groups of variables. The use of methods such as CCA provides a better overall picture of the data than two sets of p-values, obtained from standard univariate analyses, would otherwise provide, as demonstrated previously (Henderson et al., 2015; Montoliu et al., 2016).

## Results

### Physiological parameters

Dogs fed the kibble diet had a higher dry matter intake compared to dogs fed the meat diet (Table 2). Body weight of dogs fed red meat was lower at the end of the study (Table 2).

**Table 2:** Food intake and body weight. Average food intake and bodyweight in domestic dogs (*Canis familiaris*) fed either a commercially available kibbled (kibble; *n* = 8) or red meat (meat; *n* = 7) diet for nine weeks.

	Kibble	Meat	SEM	*P*-value		
				Diet	Week	Diet × week
Intake (g/d)	405	973	50.6	0.001	0.001	0.001
Dry matter (DM) intake (g DM/d)	369.8	237.4	19.1	0.001	0.001	0.001
Body weight (kg)	26.8	23.6	1.2	0.019	0.001	0.001

#### Apparent digestibility of nutrients

Apparent digestibility of DM, energy, crude fat and protein digestibility was higher in dogs fed the meat diet (Table 3). Protein and fat content of faeces was higher in dogs fed the kibble diet (Table 3).

**Table 3:** Macronutrient digestibility, volatile fatty acid concentrations and faecal health score. Apparent digestibility of dry matter, energy, crude protein and crude fat, faecal volatile fatty acid concentrations, faecal health score and faecal weight in the domestic dog (*Canis familiaris*) fed either a commercially available kibbled diet (Kibble; *n* = 8) or a red meat (Meat; *n* = 7) diet for nine weeks.

	Kibble						Meat						*P*-value		
	Week 3		Week 6		Week 9		Week 3		Week 6		Week 9		
	Mean	SEM	Mean	SEM	Mean	SEM	Mean	SEM	Mean	SEM	Mean	SEM	Diet	Time	Diet × time
*Digestibility*															
Dry matter %	82.3	1.0	83.4	1.2	81.3	1.0	78.6	3.5	91.2	1.9	97.9	0.3	0.001	0.001	0.001
Energy %	89.2	0.4	88.4	0.9	87.2	0.8	94.3	0.7	96.1	0.5	98.8	0.2	0.001	0.156	0.001
Fat %	98.4	0.1	98.3	0.1	98.1	0.1	97.8	0.3	98.9	0.1	99.7	0.1	0.015	0.001	0.001
Protein %	82.8	0.7	82.4	1.4	79.9	1.4	96.7	0.4	97.5	0.3	99.2	0.1	0.001	0.602	0.001
*Faecal parameters*															
Protein content (g DM/d)	19.1	1.0	11.5	0.9	13.7	1.1	5.1	0.5	2.5	0.3	4.7	0.8	0.001	0.001	0.001
Fat content (g DM/d)	1.6	0.1	0.9	0.1	1.2	0.1	0.6	0.1	0.3	0.0	0.4	0.1	0.001	0.001	0.003
Faecal DM (%)	35.9	1.9	32.6	0.6	31.1	0.6	56.5	3.5	47.5	6.1	43.1	2.0	0.001	0.006	0.353
Faecal Health Score	3.0	0.1	3.2	0.1	3.1	0.1	3.5	0.1	3.5	0.3	3.9	0.1	0.002	0.237	0.256
Faecal weight (g DM/d)^†^	66.4	8.8	56.9	14.0	70.4	17.6	40.1	8.6	20.9	7.5	23.9	8.3	0.001	0.026	0.05
Faecal weight (g as is/d)	184.6	4.9	183.7	3.8	227.9	4.9	68.3	6.4	43.4	4.2	56.5	4.1	0.001	0.004	0.005
*Volatile fatty acids*															
Acetate (mg/g DM)	25.9	2.5	28.3	2.8	30.4	2.7	10.1	1.9	6.9	3.2	15.4	3.6	0.001	0.157	0.558
Propionate (mg/g DM)	16.6	2.5	17.3	1.2	17.3	1.2	5.3	0.5	4.7	1.7	9.7	2.1	0.001	0.197	0.296
Butyrate (mg/g DM)	6.7	0.8	7.9	0.7	7.1	0.7	3.5	0.4	3.0	0.9	5.8	0.5	0.001	0.082	0.041
Total VFA^a^ (mg/g DM)	49.3	5.3	53.5	3.9	54.8	4.1	18.9	2.5	14.5	3.8	30.9	6.4	0.001	0.108	0.323
Isobutyrate (mg/g DM)	2.3	0.3	2.2	0.3	2.2	0.4	0.8	0.1	0.7	0.3	1.4	0.2	0.001	0.733	0.318
Valerate (mg/g DM)	0.3	0.1	0.2	0.0	0.2	0.0	0.1	0.0	0.3	0.2	0.1	0.0	0.202	0.214	0.101
Isovalerate (mg/g DM)	2.5	0.4	2.1	0.2	2.1	0.3	2.0	0.3	1.5	0.5	2.5	0.3	0.616	0.413	0.188
Caproic acid (mg/g DM)	0.05	0.0	0.1	0.0	0.1	0.0	0.0	0.0	0.1	0.0	0.0	0.0	0.050	0.422	0.373

**Notes.**

^†^Faecal Health Score is a five point scale from “Grade 1” where the faecal matter is liquid, to “Grade 5” where the faecal matter is very hard.

a Total volatile fatty acids: acetate + propionate + butyrate.

#### Faecal health score and weight

Faecal health score was affected by diet (Table 3), with dogs fed the kibble diet having a lower faecal health score than dogs fed the meat diet (*K*: 3.0 vs *M*: 3.7 (SEM 0.9); *P* = 0.002). Dogs fed the kibble diet produced more faeces compared to dogs fed meat (*K*: 198 vs *M*: 56 (SEM 14) g faeces/day; *P* = 0.001).

#### Faecal volatile fatty acids

The concentrations of acetate, propionate and butyrate were lower (*P* < 0.05) in dogs fed the meat diet (Table 3). Similarly the branched-chain fatty acids (isobutyrate, valerate and isovalerate) were lower (*P* < 0.05) in dogs fed the meat diet.

### Faecal microbial populations

In the current study, 129 genera (Appendix 2) were identified in the faeces of dogs, categorised into 50 bacterial families (Appendix 3).

#### Genera

The five most abundant genera (Appendix 2; Table 4) observed in dogs fed the kibble diet (average of weeks 3, 6 and 9) were *Peptostreptococcus* (30.2% of sequence reads), *Bacteriodes* (11.2% of sequence reads), *Prevotella* (7.2% of sequence reads), *Faecalibacterium* (6.7% of sequence reads) and *Blautia* (5.7% of sequence reads). In dogs fed the meat diet, the five most abundant genera (average of weeks 3, 6 and 9) were *Peptostreptococcus* (24.2% of sequence reads), *Fusobacterium* (12.9% of sequence reads), *Blautia* (7.2% of sequence reads), *Clostridium* (6.0% of sequence reads) and *Lactobacillus* (5.2% of sequence reads).

**Table 4:** Bacterial genera (proportion of total sequences) present in faecal samples from the domestic dog (Canis familiaris) fed either a commercially available kibbled diet or a red meat diet for nine weeks. Bacterial genera (proportion of total sequences) present in faecal samples from the domestic dog (*Canis familiaris*) fed either a commercially available kibbled diet (Kibble; *n* = 8) or a red meat (Meat; *n* = 7) diet for nine weeks. *P*-value indicates ANOVA significance of rank transformed data. Pyrosequencing of bacterial 16S rRNA gene barcoded amplicons resulted in a total of 1,490,676 sequences after denoising and chimera removal, with an average number of 25,265 sequences per sample (range = 4,603–33,179). Sequence length was on an average of 374 bp (range = 132–575 bp).

Phyla	Family	Genus	Kibble						Meat						*P*-value		
			3		6		9		3		6		9		
			Mean	SEM	Mean	SEM	Mean	SEM	Mean	SEM	Mean	SEM	Mean	SEM	Diet	Time	Diet × Week
Actinobacteria																	
	Actinomycetaceae																
		*Actinomyces*	0.00	0.00	0.00	0.00	0.00	0.00	0.01	0.00	0.01	0.00	0.01	0.003	0.002	0.232	0.102
	Bifidobacteriaceae																
		*Bifidobacterium*	0.00	0.00	0.00	0.00	0.00	0.00	0.00	0.00	0.04	0.02	0.03	0.01	0.006^*^	0.001	0.007
		*Unlc Bifidobacteriaceae*	0.00	0.00	0.00	0.00	0.00	0.00	0.00	0.00	0.02	0.00	0.01	0.003	0.006^*^	0.001	0.008
	Coriobacteriaceae																
		*Collinsella*	0.81	0.13	0.50	0.13	0.69	0.13	3.89	1.06	1.45	0.55	2.42	0.367	0.000^*^	0.001	0.003
		*Olsenella*	0.00	0.00	0.00	0.00	0.00	0.00	0.00	0.00	0.03	0.01	0.02	0.005	0.000^*^	0.000	0.000
		*Slackia*	0.18	0.03	0.10	0.03	0.12	0.03	1.04	0.32	0.74	0.18	0.49	0.06	0.000^*^	0.003	0.018
		*Uncl Coriobacteriaceae*	0.05	0.01	0.03	0.01	0.04	0.01	0.34	0.11	0.22	0.11	0.26	0.061	0.000^*^	0.006	0.037
Bacteroidetes																	
	Bacteroidaceae																
		*Bacteroides*	6.53	1.44	13.79	1.82	13.24	3.08	1.63	0.66	0.51	0.30	1.28	0.964	0.000^*^	0.128	0.000
	Porphyromonadaceae																
		*Parabacteroides*	0.00	0.00	0.00	0.00	0.00	0.00	0.31	0.19	0.38	0.13	0.38	0.204	0.002^*^	0.245	0.217
		*Porphyromonas*	0.00	0.00	0.00	0.00	0.00	0.00	0.01	0.01	0.01	0.01	0.01	0.009	0.04	0.335	0.491
	Prevotellaceae																
		*Prevotella*	7.70	3.39	5.81	3.42	8.18	3.58	0.00	0.00	0.00	0.00	0.02	0.011	0.001^*^	0.738	0.867
		*Xylanibacter*	0.02	0.01	0.01	0.01	0.02	0.01	0.00	0.00	0.00	0.00	0.00	0	0.001^*^	0.874	0.685
Firmicutes																	
	Carnobacteriaceae																
		*Carnobacterium*	0.00	0.00	0.00	0.00	0.00	0.00	0.00	0.00	0.00	0.00	0.00	0.002	0.014^*^	0.09	0.069
		*Granulicatella*	0.00	0.00	0.00	0.00	0.00	0.00	0.00	0.00	0.00	0.00	0.01	0.002	0.000	0.000	0.000
		*Uncl Carnobacteriaceae*	0.00	0.00	0.00	0.00	0.00	0.00	0.01	0.00	0.01	0.01	0.01	0.006	0.007^*^	0.505	0.350
	Enterococcaceae																
		*Enterococcus*	0.00	0.00	0.00	0.00	0.00	0.00	0.02	0.01	0.05	0.04	0.11	0.039	0.005^*^	0.126	0.002
		*Uncl Enterococcaceae*	0.00	0.00	0.00	0.00	0.00	0.00	0.00	0.00	0.01	0.00	0.02	0.006	0.009^*^	0.022	0.002
	Lactobacillaceae																
		*Lactobacillus*	0.00	0.00	0.00	0.00	0.01	0.00	0.01	0.01	0.59	0.32	14.88	7.777	0.027^*^	0.033	0.018
		*Pediococcus*	0.00	0.00	0.00	0.00	0.00	0.00	0.00	0.00	0.00	0.00	0.01	0.004	0.002^*^	0.000	0.000
		*Uncl Lactobacillaceae*	0.00	0.00	0.00	0.00	0.00	0.00	0.00	0.00	0.06	0.04	1.49	0.767	0.019^*^	0.036	0.018
	Streptococcaceae																
		*Lactococcus*	0.00	0.00	0.00	0.00	0.00	0.00	0.09	0.04	1.25	0.74	0.45	0.164	0.003^*^	0.01	0.001
	Unlc Lactobacillales		0.00	0.00	0.00	0.00	0.00	0.00	0.03	0.01	0.36	0.21	0.98	0.415	0.005^*^	0.029	0.005
	Uncl Bacilli		0.00	0.00	0.00	0.00	0.00	0.00	0.00	0.00	0.02	0.01	0.02	0.007	0.005^*^	0.008	0.003
	Clostridiaceae																
		*Clostridium*	0.19	0.12	0.10	0.05	0.06	0.01	3.40	0.80	10.68	3.22	6.10	1.525	0.000^*^	0.041	0.001
		*Uncl Clostridiaceae*	0.18	0.12	0.08	0.03	0.04	0.01	2.51	0.63	6.57	1.81	3.81	0.938	0.000^*^	0.105	0.002
	Eubacteriaceae																
		*Eubacterium*	0.23	0.07	0.42	0.14	0.26	0.08	0.03	0.01	0.05	0.03	0.03	0.011	0.000^*^	0.001	0.700
		*Unlc Eubacteriaceae*	0.00	0.00	0.00	0.00	0.00	0.00	0.01	0.00	0.01	0.00	0.01	0.003	0.002^*^	0.200	0.197
	Incertae Sedis XIII																
		*Anaerovorax*	0.01	0.00	0.01	0.00	0.00	0.00	0.03	0.03	0.03	0.03	0.04	0.025	0.033	0.975	0.981
		*Mogibacterium*	0.00	0.00	0.00	0.00	0.00	0.00	0.01	0.00	0.01	0.01	0.04	0.013	0.000^*^	0.001	0.000
	Lachnospiraceae																
		*Dorea*	0.21	0.09	0.14	0.04	0.17	0.07	0.45	0.11	0.46	0.13	0.44	0.12	0.043^*^	0.942	0.008
	Peptococcaceae																
		*Peptococcus*	0.55	0.25	0.33	0.13	0.63	0.16	2.23	0.44	2.79	1.08	1.61	0.485	0.000^*^	0.099	0.028
	Peptostreptococcaceae																
		*Peptostreptococcus*	36.69	4.75	28.28	4.75	25.74	3.31	28.32	1.86	22.61	3.40	21.70	3.436	0.012^*^	0.000	0.925
		*Sporacetigenium*	0.61	0.20	0.20	0.06	0.20	0.07	0.39	0.14	0.38	0.14	0.95	0.101	0.008^*^	0.255	0.004
		*Tepidibacter*	0.00	0.00	0.00	0.00	0.00	0.00	0.01	0.01	0.02	0.02	0.00	0.002	0.045^*^	0.519	0.262
	Ruminococcaceae																
		*Acetanaerobacterium*	0.00	0.00	0.00	0.00	0.00	0.00	0.00	0.00	0.01	0.00	0.01	0.002	0.017^*^	0.362	0.806
		*Faecalibacterium*	4.36	1.38	10.55	2.60	5.30	1.76	0.07	0.04	0.02	0.01	0.01	0.005	0.000^*^	0.004	0.004
		*Lactonifactor*	0.02	0.00	0.01	0.00	0.02	0.00	0.01	0.00	0.01	0.01	0.01	0.004	0.015^*^	0.107	0.135
		*Ruminococcus*	0.07	0.01	0.07	0.01	0.08	0.04	0.04	0.01	0.04	0.02	0.02	0.006	0.016^*^	0.336	0.288
		*Sporobacter*	0.01	0.00	0.02	0.01	0.03	0.01	0.13	0.05	0.13	0.04	0.06	0.018	0.000^*^	0.097	0.035
	Veillonellaceae																
		*Megamonas*	0.99	0.28	0.92	0.19	2.04	0.73	0.12	0.05	0.18	0.13	0.42	0.153	0.003^*^	0.096	0.528
		*Uncl Veillonellaceae*	0.65	0.21	0.72	0.11	0.88	0.17	0.23	0.13	0.12	0.04	0.10	0.038	0.011	0.000	0.004
	Erysipelotrichaceae																
		*Allobaculum*	1.68	0.28	1.34	0.27	1.16	0.29	0.34	0.08	0.78	0.30	0.22	0.066	0.000^*^	0.256	0.005
		*Catenibacterium*	0.86	0.37	0.39	0.30	0.89	0.74	0.01	0.00	0.01	0.00	0.01	0.002	0.005^*^	0.078	0.537
		*Holdemania*	0.02	0.01	0.02	0.01	0.01	0.00	0.07	0.02	0.05	0.01	0.05	0.024	0.002	0.033	0.282
		*Turicibacter*	0.76	0.28	1.32	0.45	0.96	0.32	0.00	0.00	0.00	0.00	0.00	0.004	0.01^*^	0.369	0.01
		*Uncl Erysipelotrichaceae*	0.19	0.03	0.18	0.02	0.20	0.05	0.05	0.02	0.06	0.03	0.03	0.017	0.000^*^	0.073	0.695
	Uncl Firmicutes		0.92	0.09	1.10	0.15	0.78	0.16	0.48	0.08	1.00	0.28	0.80	0.195	0.018^*^	0.003	0.289
Fusobacteria																	
	Fusobacteriaceae																
		*Cetobacterium*	0.03	0.02	0.04	0.02	0.04	0.02	0.08	0.02	0.07	0.04	0.07	0.03	0.003^*^	0.938	0.965
		*Fusobacterium*	3.96	1.38	3.63	0.93	6.71	1.72	14.64	2.57	12.84	6.21	11.29	2.967	0.000^*^	0.756	0.522
	Uncl Fusobacteriaceae		1.02	0.36	0.89	0.22	1.30	0.36	2.48	0.45	2.04	0.87	1.61	0.513	0.000^*^	0.605	0.458
Proteobacteria																	
	Succinivibrionaceae																
		*Succinivibrio*	0.74	0.38	0.28	0.14	0.49	0.32	0.00	0.00	0.00	0.00	0.00	0	0.009^*^	0.354	0.33
	Uncl Burkholderiales		0.03	0.02	0.02	0.01	0.04	0.01	0.02	0.01	0.00	0.00	0.04	0.019	0.016	0.025	0.000
Uncl Bacteria																	
	Unclassified		1.15	0.20	1.18	0.10	1.18	0.11	0.88	0.19	0.64	0.15	0.44	0.123	0.012^*^	0.482	0.007

**Notes.**

TITLE Uncl unclassified

* Significant at the 5% level in Permutation ANOVA analysis using 2,000 permutations.

**Table 5:** Bacterial families (proportion of total sequences) present in faecal samples from the domestic dog fed either a commercially available kibbled diet or a red meat diet for nine weeks. Bacterial families (proportion of total sequences) present in faecal samples from the domestic dog (*Canis familiaris*) fed either a commercially available kibbled diet (Kibble; *n* = 8) or a red meat (Meat; *n* = 7) diet for nine weeks. *P*-value indicates ANOVA significance of rank transformed data. Pyrosequencing of bacterial 16S rRNA gene barcoded amplicons resulted in a total of 1,490,676 sequences after denoising and chimera removal, with an average number of 25,265 sequences per sample (range = 4,603–33,179). Sequence length was on an average of 374 bp (range = 132–575 bp).

Family	Kibble						Meat						*P*-value		
	3		6		9		3		6		9				
	Mean	SEM	Mean	SEM	Mean	SEM	Mean	SEM	Mean	SEM	Mean	SEM	Diet	Time	Diet*Week
Clostridiaceae	0.40	0.20	0.20	0.10	0.10	0.00	6.10	1.70	13.50	4.50	9.10	2.70	0.000^*^	0.396	0.250
Coriobacteriaceae	1.00	0.20	0.60	0.20	0.80	0.20	5.40	1.20	2.00	0.90	3.40	0.40	0.000^*^	0.094	0.129
Erysipelotrichaceae	3.60	0.60	3.50	0.60	3.40	0.90	0.60	0.10	1.20	0.40	0.40	0.10	0.000^*^	0.737	0.958
Bacteroidaceae	6.50	1.40	13.80	1.80	13.20	3.10	1.60	0.80	0.70	0.40	1.50	1.10	0.000^*^	0.067	0.081
Fusobacteriaceae	5.00	1.70	4.60	1.20	8.00	2.10	18.00	3.30	19.60	7.50	14.70	3.60	0.000^*^	0.933	0.236
Peptococcaceae	0.60	0.30	0.30	0.10	0.60	0.20	2.10	0.50	2.10	1.20	1.50	0.60	0.000^*^	0.546	0.370
Ruminococcaceae	5.80	1.50	12.40	2.70	7.10	1.80	2.20	0.30	2.10	0.20	1.50	0.40	0.000^*^	0.827	0.569
Unclassified Bacilli	0.00	0.00	0.00	0.00	0.00	0.00	0.00	0.00	0.00	0.00	0.00	0.00	0.001^*^	0.074	0.048
Eubacteriaceae	0.20	0.10	0.40	0.10	0.30	0.10	0.00	0.00	0.10	0.00	0.00	0.00	0.001^*^	0.847	0.840
Bifidobacteriaceae	0.00	0.00	0.00	0.00	0.00	0.00	0.00	0.00	0.10	0.00	0.00	0.00	0.001^*^	0.043	0.022
Veillonellaceae	1.80	0.50	1.80	0.30	3.10	0.80	0.70	0.20	0.40	0.20	1.00	0.40	0.001^*^	0.086	0.318
Unclassified Bacteria	1.10	0.20	1.20	0.10	1.20	0.10	0.90	0.20	0.70	0.20	0.50	0.10	0.001^*^	0.295	0.161
Enterococcaceae	0.00	0.00	0.00	0.00	0.00	0.00	0.00	0.00	0.10	0.10	0.10	0.00	0.002^*^	0.152	0.113
Carnobacteriaceae	0.00	0.00	0.00	0.00	0.00	0.00	0.00	0.00	0.00	0.00	0.00	0.00	0.003^*^	0.122	0.087
Prevotellaceae	8.10	3.50	6.30	3.50	9.00	3.60	0.40	0.20	0.60	0.40	0.10	0.10	0.004^*^	0.903	0.844
Lachnospiraceae	2.80	0.20	2.60	0.20	3.00	0.50	4.10	0.70	4.60	0.80	3.70	0.80	0.006	0.911	0.470
Streptococcaceae	0.00	0.00	0.00	0.00	0.00	0.00	0.10	0.00	4.00	2.60	2.00	1.10	0.007	0.164	0.123
Uncl Lactobacillales	0.00	0.00	0.00	0.00	0.00	0.00	0.00	0.00	0.40	0.30	0.70	0.40	0.008^*^	0.035	0.020
Leuconostocaceae	0.00	0.00	0.00	0.00	0.00	0.00	0.00	0.00	0.00	0.00	0.00	0.00	0.009	0.015	0.007
ClostridialesIncertae Sedis XIV	5.90	0.60	5.80	0.40	5.60	0.90	8.90	0.90	5.30	0.60	7.50	1.40	0.014	0.346	0.540
Uncl Actinomycetales	0.00	0.00	0.00	0.00	0.00	0.00	0.00	0.00	0.00	0.00	0.00	0.00	0.016	0.108	0.093
Microbacteriaceae	0.00	0.00	0.00	0.00	0.00	0.00	0.00	0.00	0.00	0.00	0.00	0.00	0.021	0.683	0.443
Succinivibrionaceae	0.80	0.40	0.30	0.10	0.60	0.40	0.00	0.00	0.00	0.00	0.00	0.00	0.030^*^	0.733	0.729
Desulfovibrionaceae	0.00	0.00	0.00	0.00	0.00	0.00	0.30	0.20	0.00	0.00	0.10	0.00	0.035	0.217	0.175
Clostridiales Incertae Sedis XIII	0.00	0.00	0.00	0.00	0.00	0.00	0.10	0.10	0.10	0.00	0.20	0.10	0.039^*^	0.415	0.315
Uncl Clostridia	0.00	0.00	0.00	0.00	0.00	0.00	0.00	0.00	0.00	0.00	0.00	0.00	0.047	0.875	0.845
Uncl Firmicutes	0.90	0.10	1.10	0.20	0.80	0.20	0.50	0.10	1.00	0.40	0.70	0.20	0.057^*^	0.761	0.198
Uncl Actinobacteria	0.00	0.00	0.00	0.00	0.00	0.00	0.00	0.00	0.00	0.00	0.00	0.00	0.057	0.311	0.297
Micrococcaceae	0.00	0.00	0.00	0.00	0.00	0.00	0.00	0.00	0.00	0.00	0.00	0.00	0.058	0.545	0.569
Bacillaceae	0.00	0.00	0.00	0.00	0.00	0.00	0.00	0.00	0.10	0.10	0.00	0.00	0.068	0.570	0.579
Porphyromonadaceae	0.20	0.20	0.20	0.10	0.30	0.20	0.70	0.40	0.70	0.20	0.50	0.30	0.069	0.833	0.616
Uncl Bacillales	0.00	0.00	0.00	0.00	0.00	0.00	0.00	0.00	0.10	0.10	0.00	0.00	0.081	0.645	0.608
Lactobacillaceae	0.00	0.00	0.00	0.00	0.00	0.00	0.00	0.00	0.60	0.50	11.30	8.20	0.089^*^	0.088	0.060
Paenibacillaceae	0.00	0.00	0.00	0.00	0.00	0.00	0.00	0.00	0.00	0.00	0.00	0.00	0.101	0.837	0.558
Pseudonocardiaceae	0.00	0.00	0.00	0.00	0.00	0.00	0.00	0.00	0.00	0.00	0.00	0.00	0.114	0.480	0.977
Uncl Desulfovibrionales	0.00	0.00	0.00	0.00	0.00	0.00	0.10	0.10	0.00	0.00	0.00	0.00	0.116	0.397	0.339
Enterobacteriaceae	0.00	0.00	0.10	0.10	0.10	0.10	0.20	0.10	0.60	0.50	0.10	0.10	0.131	0.996	0.391
Alcaligenaceae	0.30	0.10	0.30	0.10	0.50	0.20	0.10	0.00	0.20	0.10	0.30	0.20	0.131	0.186	0.877
Peptostreptococcaceae	40.90	5.30	30.50	4.90	27.80	3.40	30.80	1.40	22.90	2.30	27.10	3.30	0.132	0.026	0.249
Uncl Proteobacteria	0.00	0.00	0.00	0.00	0.00	0.00	0.00	0.00	0.00	0.00	0.00	0.00	0.295	0.152	0.470
Moraxellaceae	0.00	0.00	0.00	0.00	0.00	0.00	0.00	0.00	0.00	0.00	0.00	0.00	0.369	0.142	0.767
Uncl Gammaproteobacteria	0.00	0.00	0.00	0.00	0.00	0.00	0.00	0.00	0.00	0.00	0.00	0.00	0.373	0.662	0.792
Uncl Bacteroidetes	0.10	0.00	0.10	0.00	0.10	0.00	0.10	0.10	0.10	0.10	0.00	0.00	0.509	0.860	0.075
Uncl Betaproteobacteria	0.00	0.00	0.00	0.00	0.10	0.00	0.00	0.00	0.00	0.00	0.00	0.00	0.520	0.032	0.825
Uncl Clostridiales	12.50	0.80	11.60	0.80	10.80	1.50	13.10	0.90	12.60	3.20	11.10	1.80	0.584	0.140	0.924
Anaeroplasmataceae	0.00	0.00	0.00	0.00	0.00	0.00	0.00	0.00	0.00	0.00	0.00	0.00	0.691	0.346	0.286
Uncl Bacteroidales	1.30	0.50	2.30	1.00	3.50	1.10	2.60	1.40	3.50	2.10	0.70	0.40	0.741	0.693	0.043
Uncl Burkholderiales	0.00	0.00	0.00	0.00	0.00	0.00	0.00	0.00	0.00	0.00	0.00	0.00	0.851	0.336	0.577
Uncl Fusobacteriales	0.00	0.00	0.00	0.00	0.00	0.00	0.00	0.00	0.00	0.00	0.00	0.00	0.871	0.172	0.582
Helicobacteraceae	0.00	0.00	0.00	0.00	0.00	0.00	0.00	0.00	0.00	0.00	0.00	0.00	0.949	0.309	0.538

**Notes.**

TITLE Uncl unclassified

* Significant at the 5% level in Permutation ANOVA analysis using 2,000 permutations.

Diet significantly affected the abundances of 53 genera (Table 4). In dogs fed the meat diet, the abundances of *Bacteriodes* (*K*: 9.2 vs *M*: 3.3% of sequence reads; *P* < 0.001; Permutation ANOVA *P* < 0.05), *Prevotella* (*K*: 7.4 vs *M*: 0.8% of sequence reads; *P* = 0.001; Permutation ANOVA *P* < 0.05), *Peptostreptococcus* (*K*: 27.2 vs *M*: 20.7% of sequence reads) and *Faecalibacterium* (*K*: 5.3 vs *M*: 0.3% of sequence reads; *P* < 0.001; Permutation ANOVA *P* < 0.05) were lower, compared to dogs fed the kibble diet. In contrast, the abundances of *Fusobacterium* (*K*: 4.4 vs *M*: 12.5% of sequence reads; *P* < 0.001; Permutation ANOVA *P* < 0.05), *Lactobacillus* (*K*: 0.5 vs *M*: 6.1% of sequence reads; *P* = 0.027; Permutation ANOVA *P* < 0.05), *Collinsella* (*K*: 0.7 vs *M*: 2.0% of sequence reads; *P* < 0.001; Permutation ANOVA *P* < 0.05) and *Slackia* (*K*: 0.1 vs *M*: 0.6% of sequence reads; *P* < 0.001; Permutation ANOVA *P* < 0.05) were higher in the faeces of dogs fed the meat diet (Table 4).

#### Family

The five most abundant bacterial families (Table 5) in dogs fed the kibble diet (average of weeks 3, 6 and 9) were Peptostreptococcaceae (33.1% of sequence reads), unclassified Clostridiales (11.6% of sequence reads), Bacteroidaceae (11.2% of sequence reads), Ruminococcaceae (8.4% of sequence reads) and Prevotellaceae (7.8% of sequence reads). In dogs fed the meat diet, the five most abundant bacterial families were Peptostreptococcaceae (26.9% of sequence reads), Fusobacteriaceae (17.4% of sequence reads), unclassified Clostridiales (12.3% of sequence reads), Clostridiaceae (9.6% of sequence reads) and Clostridiales Incertae Sedis XIV (7.2% of sequence reads).

Diet significantly affected (*P* < 0.05; Permutation ANOVA *P* < 0.05) the abundances of 27 families (Table 5). In dogs fed the meat diet, the abundances of Clostridiaceae (*K*: 0.2 vs *M*: 9.6% of sequence reads; *P* < 0.001; Permutation ANOVA *P* < 0.05) and Fusobacteriaceae (*K*: 5.9 vs *M*: 17.4% of sequence reads; *P* < 0.001; Permutation ANOVA *P* < 0.05) were higher. In contrast, Bacteroidaceae (11.2 vs 1.3% of sequence reads; *P* < 0.001; Permutation ANOVA *P* < 0.05), Ruminococcaceae (8.4 vs 1.9% of sequence reads; *P* < 0.001; Permutation ANOVA *P* < 0.05) and Prevotellaceae (*K*: 7.8 vs *M*: 0.4% of sequence reads; *P* = 0.004; Permutation ANOVA *P* < 0.05) were lower in dogs fed the meat diet.

#### Dataset integration

The amount of variance explained by each canonical component is indicated in Fig. S2A. When the shrunk CCA was applied to the data there was good separation between treatments but not between time-points (Fig. S2B). The correlation circle plot of the integrated data shows a number of highly correlated parameters (Fig. S3).

**Figure 2:**
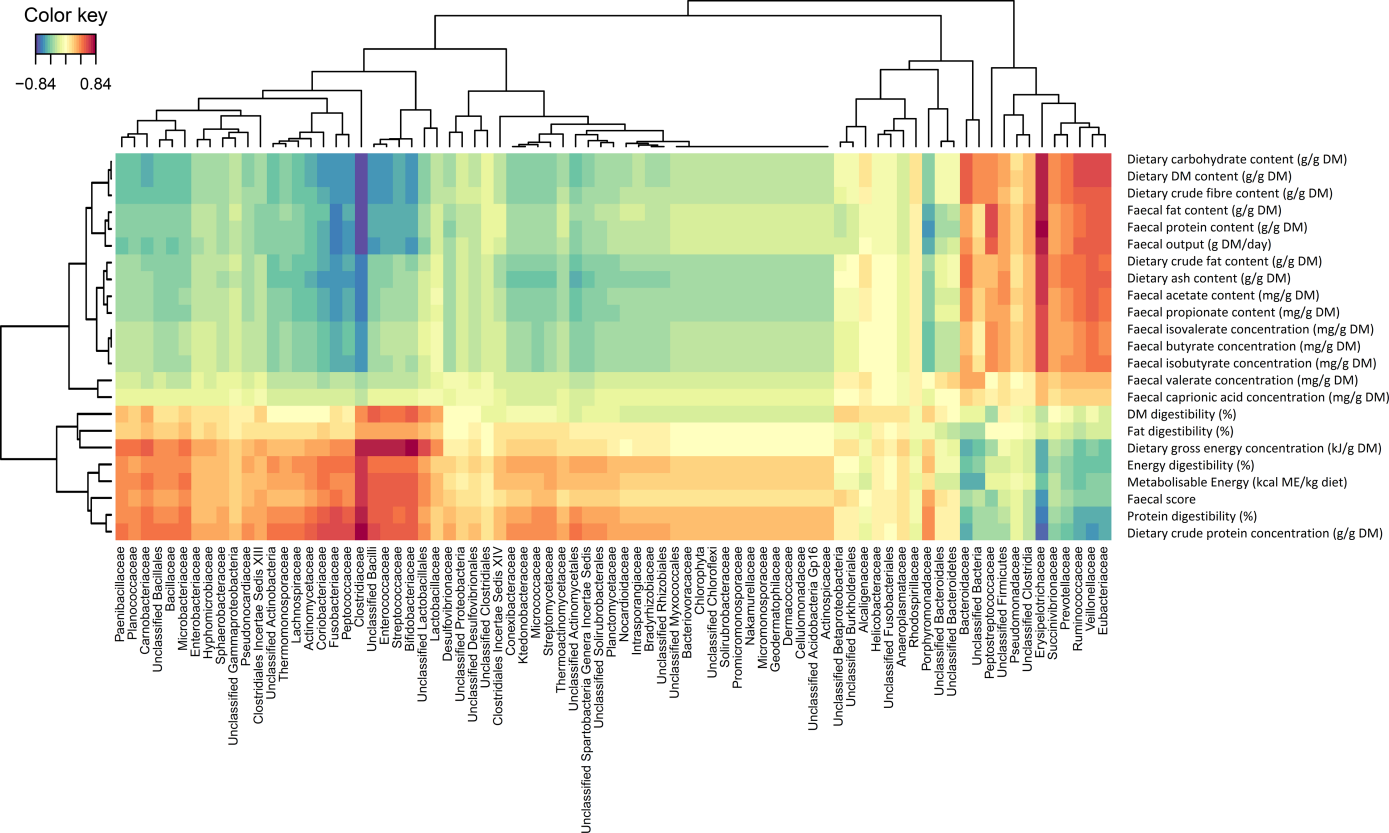
Correlation heat map describing the associations between faecal bacteria families and physiological markers of intestinal function in dogs fed both the kibbled and meat diet. Correlation heat map describing the associations between faecal bacteria levels in the dog (reported at the family level) and physiological markers of intestinal function in dogs fed both the kibbled and meat diet. Correlations greater than 0.50 were considered highly positively correlated, whereas correlations below −0.50 were considered to be highly negatively correlated. DM, Dry matter.

**Figure 3:**
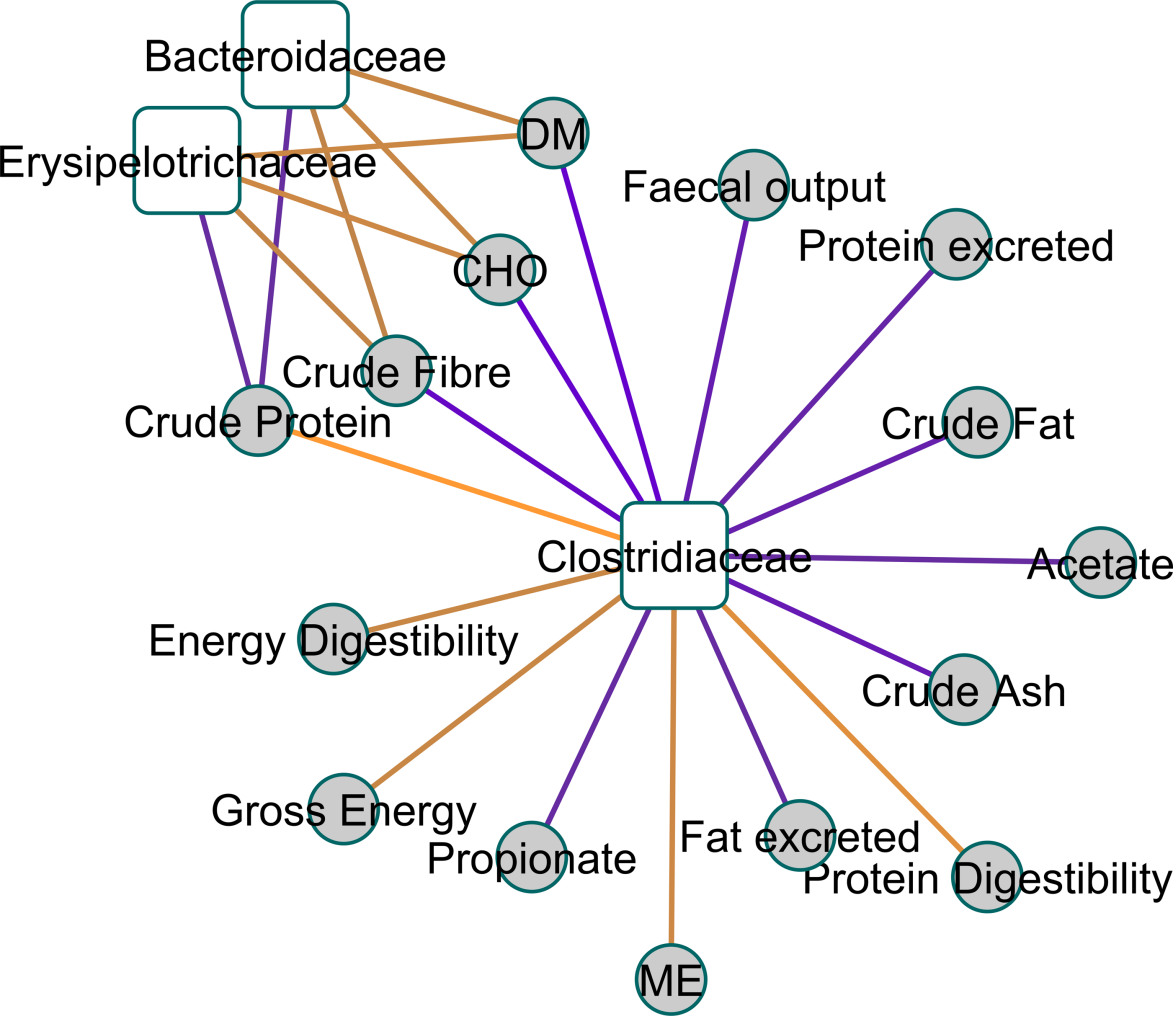
A relevance network plot (0.6 correlation cutoff) of the first two canonical dimensions of canonical correlation analysis using shrinkage. A relevance network plot (0.6 correlation cutoff) of the first two canonical dimensions of canonical correlation analysis of faecal 16S genomic DNA amplicon data with physical measurement and metabolomics data using shrinkage reveals *Clostridiaceae* as a central node.

Figure 2 shows a Clustered Image Map, highlighting the relationships between Clostridiaceae and the physiological parameters measured. A network plot (canonical correlation cut-off of 0.6 on 3 dimensions; Fig. 3) shows Clostridiaceae, Erysipelotrichaceae and Bacteroidaceae as central to the relationships between microbiota and physiological parameters. A high positive correlation was observed between Clostridiaceae and crude dietary protein content and crude protein digestibility (>0.84), and gross dietary energy content and faecal score (>0.77) and energy digestibility (>0.70), and a weaker positive correlation with fat digestibility (0.35), Fig. 2. In contrast, Clostridiaceae were negatively correlated with total protein and fat excreted and faecal output (<−0.70) and metabolites of protein digestion (e.g., isobutryrate and isovalerate; <−0.63), and dietary fat content (<−0.70). Negative correlations between Clostridiaceae and the levels of acetate, propionate and butyrate were also observed (−0.56 to −0.63). A more detailed analysis shows that within the Clostridiaceae family, *Clostridium* and *unclassified Clostridiaceae* were positively correlated to faecal dry matter (>0.66) and crude protein content of the diet and negatively correlated with crude fat content (<−0.66) of the diet (Figs. S3 and Fig. S4).

Erysipelotrichaceae were positively correlated with total protein and fat excreted (>0.84), metabolites of protein digestion (e.g., isobutryrate and isovalerate; >0.70), fat content of the diet and faecal output (>0.77), Fig. 2. Positive correlations with the levels of aceate, propionate and butyrate were also observed (0.63–0.70). Erysipelotrichaceae were negatively correlated with crude protein content of the diet and crude protein digestibility (<−0.77), faecal health score (<−0.70), gross energy content of the diet (<−0.63), energy digestibility (>−0.63) and fat digestibility (>−0.14) were observed (Fig. 2). A more detailed analysis shows that the correlations between genera within the Erysipelotrichaceae family (namely *Allobaculum*, *Catenibacterium*, *Holdemania*, *Turibacter* and *unclassified Erysipelotrichaceae*) had weaker associations with the parameters measured (Fig. S4).

Bacteroidaceae showed positive correlations with dietary carbohydrate (>0.56), crude fibre (>0.56) and dry matter (>0.56) content and faecal output (>0.35). In contrast Bacteroidaceae were negatively correlated with the crude protein (<−0.63) and gross energy content of the diet (<−0.63) and faecal health score (<−0.35). *Bacteriodes* was the only genus associated with Bacteriodaceae, and a more detailed analysis shows a strong positive correlation with valerate (Fig. S3) and the levels of protein and fat in the faeces (Fig. S3).

Bifidobacteriaceae showed a strong positive correlation to fat digestibility (>0.84; Fig. 2) and a negative correlation to faecal output and crude fibre and carbohydrate content (<−0.63). Lactobacillaceae showed only weak correlations to fat digestibility (>0.42) whereas Ruminococcaceae was positively correlated to the content of carbohydrate and dry matter in the diet (>0.63; Fig. 2).

## Discussion

There is a growing understanding of the importance of the intestinal microbiota on health, both in humans and other animals. To date, the interpretation of changes in faecal microbiota in pets has largely been based on changes observed in omnivorous humans and rodents, and the mechanisms by which microbiota influence health in carnivorous companion animals is poorly understood in comparison. Integration of the changes in microbial composition and phenotypic data such as dietary information and physiological effects (e.g., nutrient digestibility, metabolite production) is required to elucidate the effects of changes in microbial populations on optimal nutrition for pets (Deng & Swanson, 2015). Indeed a recent study has shown that the microbiota involved in inflammatory bowel disease (IBD) in the dog and human differ, with *Fusobacterium* (implicated in human IBD) being associated with a healthy microbiota in the dog (Vazquez-Baeza et al., 2016). However, such integrated analyses of datasets remain uncommon in healthy animals. In the current study we showed that Clostridiaceae, Erysipelotrichaceae and Bacteroidaceae may play central roles in the relationships between microbiota, macronutrient composition and digestibility, and faecal health score and faecal weight. These three families have all been identified in the faecal microbiota of healthy dogs (Kerr et al., 2013; Garcia-Mazcorro et al., 2012; Garcia-Mazcorro et al., 2011; Gagne et al., 2013; Handl et al., 2011; Panasevich et al., 2015).

In the current study, Clostridiaceae appears to be the central node in the relationship between microbiota, macronutrient composition and digestibility, and faecal health score and faecal weight. We observed an increase in Clostridiaceae in dogs fed meat diets, driven by increases in both *Clostridium* and unclassified *Clostridiaceae*. Clostridiaceae is a highly diverse family, encompassing genera that are important in nutrient digestibility and those that are considered to be pathogenic (Rajilic-Stojanovic & De Vos, 2014). We observed a positive correlation between Clostridiaceae and dietary protein content and protein digestibility, and a negative correlation with faecal protein content. This suggests that Clostridiaceae have a role in the metabolism of protein in the intestinal tract or that they are able to thrive in a system lacking dietary carbohydrates. Although no studies have addressed the impact of dietary protein levels and microbiota composition in dogs, in cats both Clostridiaceae and *Clostridium* are higher with high protein diets (Hooda et al., 2013; Bermingham et al., 2013a; Bermingham et al., 2013c). Surprisingly few studies have investigated the impacts of dietary protein on microbial composition in humans (Graf et al., 2015) with the impacts of dietary protein on *Clostridium* not reported (Russell et al., 2011). Despite Clostridiaceae being associated with increased dietary fibre in rodent models (Nagy-Szakal et al., 2013), we observed a negative correlation with crude fibre and parameters relating to fibre/carbohydrate metabolism (e.g., VFA concentrations) in the current study. This suggests that the Clostridiaceae perform a different role in the large bowel of the dog compared to the rat; in the rat they respond to dietary carbohydrates, but in the dog they are stimulated by dietary protein. This is consistent with observations in previous dog studies showing no effect of fibre (Panasevich et al., 2015; Beloshapka et al., 2013) on the abundances of Clostridiaceae, except at very high levels of fibre inclusion in the diet (6%) (Panasevich et al., 2015).

Some *Clostridium* spp. are thought to be pathogenic, however it is likely that the role of Clostridiaceae in the intestinal health of the dog is complex. For example, while Clostridiaceae levels were similar in healthy dogs and dogs with non-haemorrhagic diarrhoea (Suchodolski et al., 2012), *Clostridia* were increased in dogs with non-haemorrhagic diarrhoea (Suchodolski et al., 2012) and acute diarrhoea (Guard et al., 2015). In the current study, Clostridiaceae had a positive correlation with faecal health score (i.e., faeces were firmer) and a negative correlation with faecal output (i.e., less faecal output), suggesting that those Clostridiaceae members that increase in abundance when meat diets are fed are not detrimental to the health of the dog.

Erysipelotrichaceae also appear to be important bacteria in the digestive tract of the dog. Here we observed an increase in Erysipelotrichaceae in dogs fed the kibble diet, driven by increased abundances of *Allobaculum, Catenibacterium, Turibacter* and *Unclassified Erysipelotrichaceae.* In contrast, the abundance of *Holdemania* was decreased in dogs fed the kibbled diet.

As with Clostridiaceae, Erysipelotrichaceae are a diverse family of microbiota (Rajilic-Stojanovic & De Vos, 2014). In humans and in rodent models, Erysipelotrichaceae have been associated with high fat diets (Martinez et al., 2009; Fleissner et al., 2010), consistent with the strong positive correlation with dietary fat content and negative correlation with fat digestibility observed in the current study. We also observed that Erysipelotrichaceae were positively correlated with a number of markers associated with carbohydrate digestion, including dietary carbohydrate and fibre content and VFA production, which is in agreement with previous results showing increased Erysipelotrichaceae in rodents fed resistant starch (Young et al., 2013) or non-fermentable fibre (Cox et al., 2013). We also observed a negative correlation between Erysipelotrichaceae and markers of protein metabolism (e.g., protein content of the diet, apparent protein digestibility) in the intestinal tract. Erysipelotrichaceae appear to be unaffected by protein and/or fibre source in the dog (Kerr et al., 2013; Panasevich et al., 2015; Beloshapka et al., 2013) and cat (Hooda et al., 2013; Bermingham et al., 2013c). However, the abundances of *Allobaculum* and *Unclassified Erysipelotrichaceae* were higher in cats fed diets containing moderate-high levels of dietary protein suggesting that they may be affected by dietary protein (Bermingham et al., 2013c).

In humans, Erysipelotrichaceae have been associated with a number of diseases such as inflammation-related intestinal disease and metabolic disorders (Kaakoush, 2015). While the levels of different members of the Erysipelotrichaceae family such as *Allobaculum*, *Catenibacterium* and *Turibacter* were similar between healthy dogs and those with intestinal disease (diarrhoea or IBD) (Suchodolski et al., 2012; Guard et al., 2015), the levels of Erysipelotrichaceae were higher in healthy dogs compared to dogs with acute haemorrhagic diarrhoea (Suchodolski et al., 2012). Recently, members of the Erysipelotrichaceae family such as *Allobaculum* and *Catenibacterium* have been identified as part of a healthy microbiota in the dog, compared to dogs with IBD (Vazquez-Baeza et al., 2016). The role of *Holdemania* in intestinal health is unclear, with only one published study citing levels of *Holdemania* in the faecal microbiome of the dog similar to those observed in the current study (Panasevich et al., 2015).

Bacteroidaceae also appear to play a role in the digestion of nutrients in dogs. *Bacteroides* was the only genus associated with Bacteroidaceae identified in the current study, with increased abundance in dogs fed the kibble diet. Bacteroidaceae as with other members of the *Bacteriodetes* produce a number of VFA including succinate, acetate and propionate (Rajilic-Stojanovic & De Vos, 2014). As with Erysipelotrichaceae, the abundances of both Bacteroidaceae and *Bacteriodes* in dogs appear to be unaffected by protein and/or fibre source (Kerr et al., 2013; Panasevich et al., 2015; Beloshapka et al., 2013). *Bacteroides* spp. are well adapted to the intestinal environment and are known to use a wide variety of substrates for growth including simple and complex polysaccharides (Salyers et al., 1977), mucin glycoproteins (Mahowald et al., 2009), and proteins (Hughes, Magee & Bingham, 2000). In particular, *Bacteroides* spp. are associated with carbohydrate metabolism in rodents and humans (Vazquez-Baeza et al., 2016), a result consistent with their high correlation with dietary carbohydrate and crude fibre content in the current study. In kittens, the abundance of *Bacteriodes* is unaffected by a low carbohydrate-high protein diet compared to high carbohydrate-moderate protein diet (Bermingham et al., 2013a), however in older cats fed similar diets, *Bacteriodes* were significantly higher in cats fed high carbohydrate-moderate protein diets (Bermingham et al., 2013c). In the current study, *Bacteriodes* were negatively associated with crude protein content of the diets, consistent with a greater role in carbohydrate digestion than in protein digestion in the dog. This suggests that *Bacteriodes* may undertake a different function in cats and dogs, with their function in dogs similar to that in rodents and humans. This is an example of the need for species-specific data when investigating the effects of diet on the microbiota.

We found a weak correlation between *Bacteriodes* and markers of faecal health (faecal output and faecal health score). While some members of *Bacteroides* have been implicated in poor health outcomes for humans (Wexler, 2007), the abundance of *Bacteroides* was unaffected by intestinal disease in the dog (diarrhoea or inflammatory bowel disease) (Suchodolski et al., 2012; Guard et al., 2015) and is implicated in a healthy microbiota in the dog (Vazquez-Baeza et al., 2016).

Studies in humans currently focus on the effect of dietary ingredients on *Bifidobacteria* (Bifidobacteriaceae), *Lactobacillus* (Lactobacillaceae), and increasingly, *Faecalibacterium* (Ruminococcaceae), because these are thought to improve intestinal health in omnivores (Turroni, Van Sinderen & Ventura, 2009), in part through their roles in butyrate production. While bifidobacteria and lactobacilli do not produce butyrate themselves, butyrate is produced by other bacteria feeding on acetate (e.g., *Faecalibacterium*) and lactate produced by bifidobacteria and lactobacilli. As well as being an eventual end product from carbohydrate fermentation, butyrate can be formed from protein sources, with *Fusobacterium* spp. associated with this role (Vital et al., 2015). For this reason, *Bifidobacteria* and *Lactobacillus* have also been the focus in studies investigating the impacts of diet on pet health. In the current study, Bifidobacteriaceae, in particular *Bifidobacterium,* were increased in dogs fed the meat-based diet. While overall levels of Lactobacillaceae were similar between dietary treatments (*P* = 0.94), dogs fed the meat diet had increased faecal levels of *Lactobacillus*. In addition, Bifidobacteriaceae and Lactobacillaceae (which includes *Lactobacillus* and * Unclassified Lactobacillaceae*) were poorly correlated with VFA production. *Ruminococcaceae*, of which *Faecalibacterium* was the dominant genus identified, were positively (albeit relatively weakly, >0.42) correlated to butyrate concentrations, with Erysipelotrichaceae having the strongest correlation with faecal butyrate concentrations (<0.77). This suggests bacterial interactions may be different in dogs compared to rodents and humans. Indeed a recent study has highlighted that butyrate is formed via different metabolic pathways in carnivores compared to omnivores, specifically via butyrate kinase genes coming from *Clostridium perfringens* in carnivores (Vital et al., 2015). Interestingly, both *Fusobacterium* spp. and *Clostridium* spp. were negatively correlated with butyrate concentrations in the current study, suggesting that further investigation is required.

We observed a higher abundance of Ruminococcaceae in dogs fed the kibble diet; *Faecalibacterium* was the most abundant genera associated with this bacterial family. Reduced *Faecalibacterium* has typically been associated with acute diarrhoea in cats (Suchodolski et al., 2015) and dogs (Guard et al., 2015), and with IBD in dogs (Suchodolski et al., 2012; Honneffer, Minamoto & Suchodolski, 2014). However, dogs fed the meat diet did not show any signs of intestinal upset, as indicated by the improved faecal health score and reduced faecal weight. Indeed, Ruminococcaceae were positively correlated with faecal output and negatively correlated with faecal health score, suggesting that as Ruminococcaceae increase faecal quality may be reduced.

We used several parameters to assess intestinal health in the dog including macronutrient digestibility and faecal quality (faecal weight and consistency, as measured by faecal health score) and VFA production. We observed improved apparent protein and energy digestibility, reduced faecal weight and better faecal consistency in dogs fed the meat diet, but lower VFA production. Apparent digestibility of energy and crude protein was also higher in dogs fed the meat diet. This is in agreement with previous studies in cats that have shown that both raw and cooked beef diets result in higher apparent total tract dry matter, crude protein, crude fat and gross energy digestibility compared to extruded (kibbled) diets (Kerr et al., 2012).

## Conclusions

To our knowledge, this is the first comprehensive study undertaken to investigate the relationship between physiological parameters such as macronutrient digestibility and faecal health score, and faecal microbial composition in dogs. We have shown that the microbiota changes in response to diet in dogs, with Clostridiaceae, Erysipelotrichaceae and Bacteroidaceae apparently central to the relationships between microbiota and intestinal health. The data suggest that when interpreting changes in microbial composition in relation to diet, comparisons to other species may not be valid. The data also provide a basis for future studies which may further characterise the functional role of the microbiota and how they interact with diet to influence health. The understanding gained by such studies could lead to a new definition of optimal nutrition for carnivorous pets, and a range of products based on that definition.

##  Supplemental Information

10.7717/peerj.3019/supp-1 Supplemental Information 1 Supplementary tables for key bacterial families ( *Clostridiaceae, Erysipelotrichaceae* and * Bacteroidaceae*) are related to the digestion of protein and energy in dogs

10.7717/peerj.3019/supp-2 Supplemental Information 2 Schematic showing the study design, samples taken and statistical analysis

10.7717/peerj.3019/supp-3 Supplemental Information 3 Screenplot of canonical correlation analysis of faecal 16S genomic DNA amplicon data with physical measurement using shrinkage The first dimension has the highest canonical correlation.

10.7717/peerj.3019/supp-4 Supplemental Information 4 Variate plots of the first two canonical dimensions of canonical correlation analysis of faecal 16S genomic DNA amplicon data with physical measurement using shrinkage The first dimension separates diet well. The time points are not separated well in either dimension. Red text indicates dogs fed the kibble diet at 3, 6 or 9 weeks and black text indicates dogs fed the meat diet at at 3, 6 or 9 weeks.

10.7717/peerj.3019/supp-5 Supplemental Information 5 The correlation circle plot of the first two canonical dimensions of canonical correlation analysis of faecal 16S genomic DNA amplicon data Includes physical measurement and metabolomics data using shrinkage showing a number of highly correlated parameters including positive correlation between Clostridiaceae and physical measurement data.

10.7717/peerj.3019/supp-6 Supplemental Information 6 A network plot of the first two canonical dimensions of regularised canonical correlation analysis of faecal 16S genomic DNA amplicon genus data Includes physical measurement and metabolomics data (0.6 cutoff) revealing Clostridiaceae as a central node in dogs fed both the meat and kibbled diets.

10.7717/peerj.3019/supp-7 Supplemental Information 7 Correlation heat map Correlation heat map describing the associations between Clostridiaceae (*Clostridium* and *Unclassified Clostridiaceae*), Erysipelotrichaceae (*Allobaculum*, *Catenibacterium*, *Holdemania*, *Turicibacter* and *Unclassified Erysipelotrichaceae*) and Bacteriodaceae (*Bacteroides*) levels in dogs and physiological markers of intestinal function fed both the kibbled and meat diet. Correlations greater than 0.50 were considered highly positively correlated, whereas correlations below −0.50 were considered to be highly negatively correlated.

## Additional Information and Declarations

### Data Availability

The following information was supplied regarding data availability: Raw sequences (demultiplexed sff files) will be publically available from the NCBI Sequence Read Archive database (SRA) prior to publication. The assigned accession ID is PRJNA341403. http://www.ncbi.nlm.nih.gov/bioproject/341403.

## References

[ref-1] Association of American Feed Control Officials (2016). 2016 official publication.

[ref-2] Beloshapka AN, Dowd SE, Suchodolski JS, Steiner JM, Duclos L, Swanson KS (2013). Fecal microbial communities of healthy adult dogs fed raw meat-based diets with or without inulin or yeast cell wall extracts as assessed by 454 pyrosequencing. FEMS Microbiology Ecology.

[ref-3] Bermingham EN, Kittelmann S, Young W, Kerr KR, Swanson KS, Roy NC, Thomas DG (2013a). Post-weaning diet affects faecal microbial composition but not selected adipose gene expression in the cat (*Felis catus*). PLOS ONE.

[ref-4] Bermingham EN, Weidgraaf K, Hekman M, Roy NC, Tavendale MH, Thomas DG (2013b). Seasonal and age effects on energy requirements in domestic short-hair cats (*Felis catus*) in a temperate environment. Journal of Animal Physiology and Animal Nutrition.

[ref-5] Bermingham EN, Young W, Kittelmann S, Kerr KR, Swanson KS, Roy NC, Thomas DG (2013c). Dietary format alters fecal bacterial populations in the domestic cat (*Felis catus*). MicrobiologyOpen.

[ref-6] Bosch G, Hagen-Plantinga EA, Hendriks WH (2015). Dietary nutrient profiles of wild wolves: insights for optimal dog nutrition?. British Journal of Nutrition.

[ref-7] Buffington CA (2008). Dry foods and risk of disease in cats. Canadian Veterinary Journal.

[ref-8] Caporaso JG, Kuczynski J, Stombaugh J, Bittinger K, Bushman FD, Costello EK, Fierer N, Pena AG, Goodrich JK, Gordon JI, Huttley GA, Kelley ST, Knights D, Koenig JE, Ley RE, Lozupone CA, McDonald D, Muegge BD, Pirrung M, Reeder J, Sevinsky JR, Turnbaugh PJ, Walters WA, Widmann J, Yatsunenko T, Zaneveld J, Knight R (2010). QIIME allows analysis of high-throughput community sequencing data. Nature Methods.

[ref-9] Cox LM, Cho I, Young SA, Anderson WHK, Waters BJ, Hung S-C, Gao Z, Mahana D, Bihan M, Alekseyenko AV, Methé BA, Blaser MJ (2013). The nonfermentable dietary fiber hydroxypropyl methylcellulose modulates intestinal microbiota. The FASEB Journal.

[ref-10] Deng P, Swanson KS (2015). Gut microbiota of humans, dogs and cats: current knowledge and future opportunities and challenges. British Journal of Nutrition.

[ref-11] Duran-Pinedo AE, Frias-Lopez J (2015). Beyond microbial community composition: functional activities of the oral microbiome in health and disease. Microbes and Infection.

[ref-12] Fleissner CK, Huebel N, Abd El-Bary MM, Loh G, Klaus S, Blaut M (2010). Absence of intestinal microbiota does not protect mice from diet-induced obesity. British Journal of Nutrition.

[ref-13] Gagne JW, Wakshlag JJ, Simpson KW, Dowd SE, Latchman S, Brown DA, Brown K, Swanson KS, Fahey Jr GC (2013). Effects of a synbiotic on fecal quality, short-chain fatty acid concentrations, and the microbiome of healthy sled dogs. BMC Veterinary Research.

[ref-14] Garcia-Mazcorro JF, Dowd SE, Poulsen J, Steiner JM, Suchodolski JS (2012). Abundance and short-term temporal variability of fecal microbiota in healthy dogs. MicrobiologyOpen.

[ref-15] Garcia-Mazcorro JF, Lanerie DJ, Dowd SE, Paddock CG, Grutzner N, Steiner JM, Ivanek R, Suchodolski JS (2011). Effect of a multi-species synbiotic formulation on fecal bacterial microbiota of healthy cats and dogs as evaluated by pyrosequencing. FEMS Microbiology Ecology.

[ref-16] German AJ (2006). The growing problem of obesity in dogs and cats. Journal of Nutrition.

[ref-17] González I, Cao KAL, Davis MJ, Déjean S (2012). Visualising associations between paired ‘omics’ data sets. BioData Mining.

[ref-18] Graf D, Di Cagno R, Fak F, Flint HJ, Nyman M, Saarela M, Watzl B (2015). Contribution of diet to the composition of the human gut microbiota. Microbial Ecology in Health and Disease.

[ref-19] Guard BC, Barr JW, Reddivari L, Klemashevich C, Jayaraman A, Steiner JM, Vanamala J, Suchodolski JS (2015). Characterization of microbial dysbiosis and metabolomic changes in dogs with acute diarrhea. PLOS ONE.

[ref-20] Handl S, Dowd SE, Garcia-Mazcorro JF, Steiner JM, Suchodolski JS (2011). Massive parallel 16S rRNA gene pyrosequencing reveals highly diverse fecal bacterial and fungal communities in healthy dogs and cats. FEMS Microbiology Ecology.

[ref-21] Hartstra AV, Bouter KE, Backhed F, Nieuwdorp M (2015). Insights into the role of the microbiome in obesity and type 2 diabetes. Diabetes Care.

[ref-22] He C, Shan Y, Song W (2015). Targeting gut microbiota as a possible therapy for diabetes. Nutrition Research.

[ref-23] Henderson G, Cox F, Ganesh S, Jonker A, Young W, Janssen PH, Global Rumen Census Collaborators (2015). Rumen microbial community composition varies with diet and host, but a core microbiome is found across a wide geographical range. Scientific Reports.

[ref-24] Hervé M (2014).

[ref-25] Honneffer JB, Minamoto Y, Suchodolski JS (2014). Microbiota alterations in acute and chronic gastrointestinal inflammation of cats and dogs. World Journal of Gastroenterology.

[ref-26] Hooda S, Vester Boler BM, Kerr KR, Dowd SE, Swanson KS (2013). The gut microbiome of kittens is affected by dietary protein:carbohydrate ratio and associated with blood metabolite and hormone concentrations. British Journal of Nutrition.

[ref-27] Hughes R, Magee EA, Bingham S (2000). Protein degradation in the large intestine: relevance to colorectal cancer. Current Issues in Intestinal Microbiology.

[ref-28] Kaakoush NO (2015). Insights into the role of Erysipelotrichaceae in the human host. Frontiers in Cellular and Infection Microbiology.

[ref-29] Kerr KR, Forster G, Dowd SE, Ryan EP, Swanson KS (2013). Effects of dietary cooked navy bean on the fecal microbiome of healthy companion dogs. PLOS ONE.

[ref-30] Kerr KR, Vester Boler BM, Morris CL, Liu KJ, Swanson KS (2012). Apparent total tract energy and macronutrient digestibility and fecal fermentative end-product concentrations of domestic cats fed extruded, raw beef-based, and cooked beef-based diets. Journal of Animal Science.

[ref-31] Mahowald MA, Rey FE, Seedorf H, Turnbaugh PJ, Fulton RS, Wollam A, Shah N, Wang C, Magrini V, Wilson RK, Cantarel BL, Coutinho PM, Henrissat B, Crock LW, Russell A, Verberkmoes NC, Hettich RL, Gordon JI (2009). Characterizing a model human gut microbiota composed of members of its two dominant bacterial phyla. Proceedings of the National Academy of Sciences of the United States of America.

[ref-32] Martinez I, Wallace G, Zhang C, Legge R, Benson AK, Carr TP, Moriyama EN, Walter J (2009). Diet-induced metabolic improvements in a hamster model of hypercholesterolemia are strongly linked to alterations of the gut microbiota. Applied and Environmental Microbiology.

[ref-33] Montoliu I, Cominetti O, Boulangé CL, Berger B, Siddharth J, Nicholson J, Martin FPJ (2016). Modeling longitudinal metabonomics and microbiota interactions in C57BL/6 mice fed a high fat diet. Analytical Chemistry.

[ref-34] Nagy-Szakal D, Hollister EB, Luna RA, Szigeti R, Tatevian N, Smith CW, Versalovic J, Kellermayer R (2013). Cellulose supplementation early in life ameliorates colitis in adult mice. PLOS ONE.

[ref-35] Panasevich MR, Kerr KR, Dilger RN, Fahey GC, Guérin-Deremaux L, Lynch GL, Wils D, Suchodolski JS, Steer JM, Dowd SE, Swanson KS (2015). Modulation of the faecal microbiome of healthy adult dogs by inclusion of potato fibre in the diet. British Journal of Nutrition.

[ref-36] R Core Team (2015).

[ref-37] Rajilic-Stojanovic M, De Vos WM (2014). The first 1000 cultured species of the human gastrointestinal microbiota. FEMS Microbiology Reviews.

[ref-38] Russell WR, Gratz SW, Duncan SH, Holtrop G, Ince J, Scobbie L, Duncan G, Johnstone AM, Lobley GE, Wallace RJ, Duthie GG, Flint HJ (2011). High-protein, reduced-carbohydrate weight-loss diets promote metabolite profiles likely to be detrimental to colonic health. American Journal of Clinical Nutrition.

[ref-39] Salyers AA, Vercellotti JR, West SE, Wilkins TD (1977). Fermentation of mucin and plant polysaccharides by strains of Bacteroides from the human colon. Applied and Environmental Microbiology.

[ref-40] Suchodolski JS, Foster ML, Sohail MU, Leutenegger C, Queen EV, Steiner JM, Marks SL (2015). The fecal microbiome in cats with diarrhea. PLOS ONE.

[ref-41] Suchodolski JS, Markel ME, Garcia-Mazcorro JF, Unterer S, Heilmann RM, Dowd SE, Kachroo P, Ivanov I, Minamoto Y, Dillman EM, Steiner JM, Cook AK, Toresson L (2012). The fecal microbiome in dogs with acute diarrhea and idiopathic inflammatory bowel disease. PLOS ONE.

[ref-42] Swanson KS, Dowd SE, Suchodolski JS, Vester BM, Barry KA, Nelson KE, Torralba M, Henrissat B, Coutinho PM, Cann IK, White BA, Fahey Jr GC (2011). Phylogenetic and gene-centric metagenomics of the canine intestinal microbiome reveals similarities with humans and mice. ISME Journal.

[ref-43] Turroni F, Van Sinderen D, Ventura M (2009). Bifidobacteria: from ecology to genomics. Frontiers in Bioscience.

[ref-44] Vazquez-Baeza Y, Hyde ER, Suchodolski JS, Knight R (2016). Dog and human inflammatory bowel disease rely on overlapping yet distinct dysbiosis networks. Nature Microbiology.

[ref-45] Verbrugghe A, Hesta M, Daminet S, Janssens GP (2012). Nutritional modulation of insulin resistance in the true carnivorous cat: a review. Critical Reviews in Food Science and Nutrition.

[ref-46] Vital M, Gao J, Rizzo M, Harrison T, Tiedje JM (2015). Diet is a major factor governing the fecal butyrate-producing community structure across Mammalia, Aves and Reptilia. ISME Journal.

[ref-47] Wexler HM (2007). Bacteroides: the good, the bad, and the nitty-gritty. Clinical Microbiology Reviews.

[ref-48] Wronkowska M, Soral-Śmietana M, Krupa U, Biedrzycka E (2006). *In vitro* fermentation of new modified starch preparations—changes of microstructure and bacterial end-products. Enzyme and Microbial Technology.

[ref-49] Young W, Egert M, Bassett SA, Bibiloni R (2015). Detection of sialic acid-utilising bacteria in a caecal community batch culture using RNA-based stable isotope probing. Nutrients.

[ref-50] Young W, Roy NC, Lee J, Lawley B, Otter D, Henderson G, Tannock GW (2013). Bowel microbiota moderate host physiological responses to dietary konjac in weanling rats. Journal of Nutrition.

