# Supplementary figures and images for: Key bacterial families (Clostridiaceae, Erysipelotrichaceae and Bacteroidaceae) are related to the digestion of protein and energy in dogs
Source: PeerJ. 2017 Mar 2;5:e3019. doi: 10.7717/peerj.3019 (PMC5337088; doi:10.7717/peerj.3019)

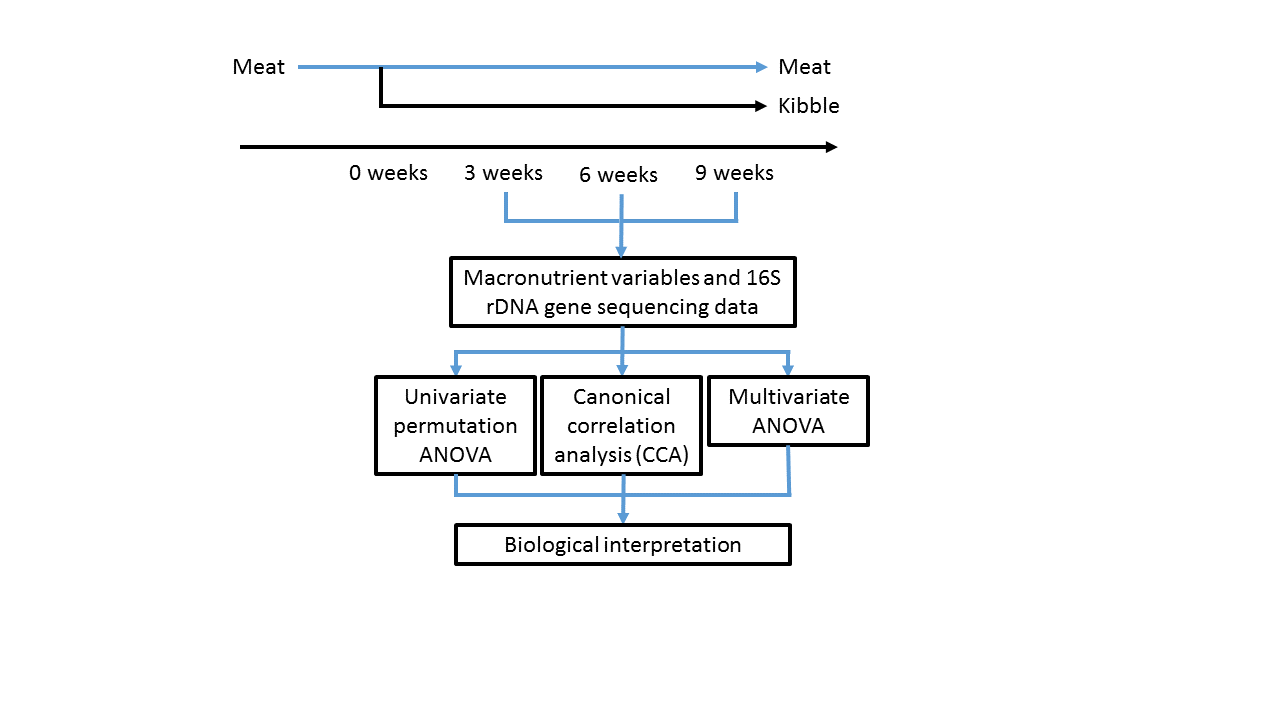

Supplement: Supplemental Information 2 [file peerj-05-3019-s002.png]

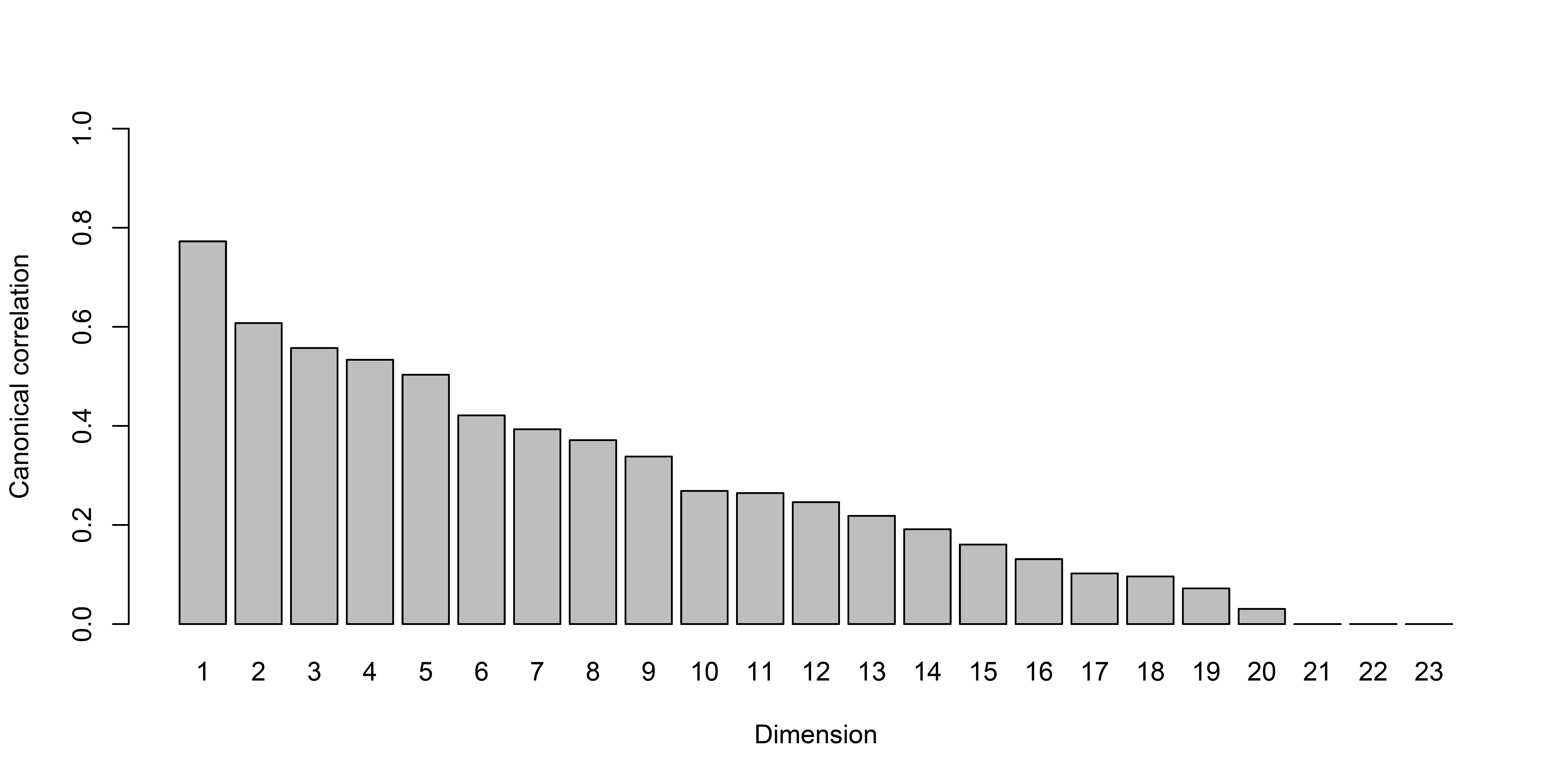

Supplement: Supplemental Information 3 — The first dimension has the highest canonical correlation. [file peerj-05-3019-s003.png]

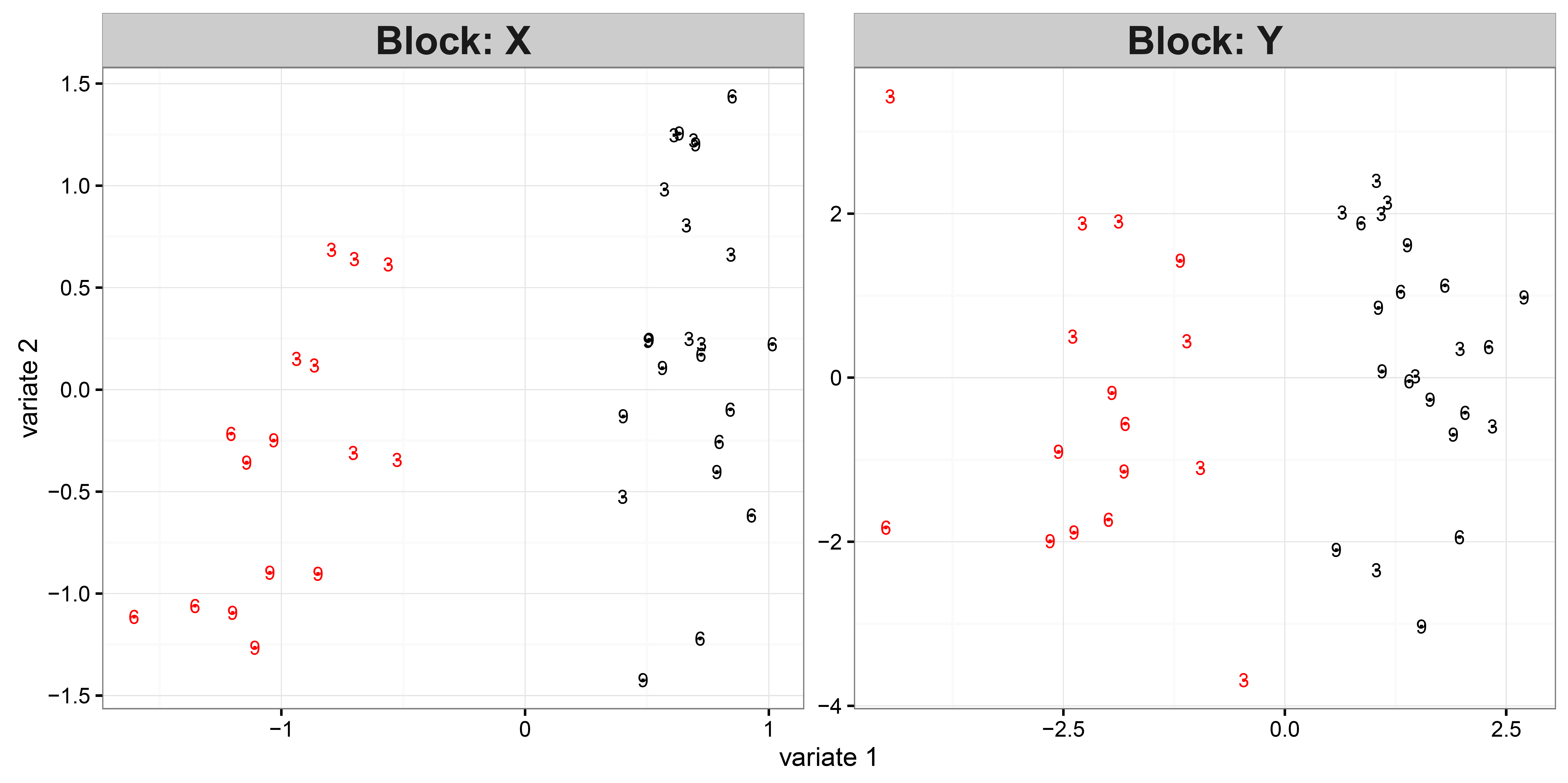

Supplement: Supplemental Information 4 — The first dimension separates diet well. The time points are not separated well in either dimension. Red text indicates dogs fed the kibble diet at 3, 6 or 9 weeks and black text indicates dogs fed the meat diet at at 3, 6 or 9 weeks. [file peerj-05-3019-s004.png]

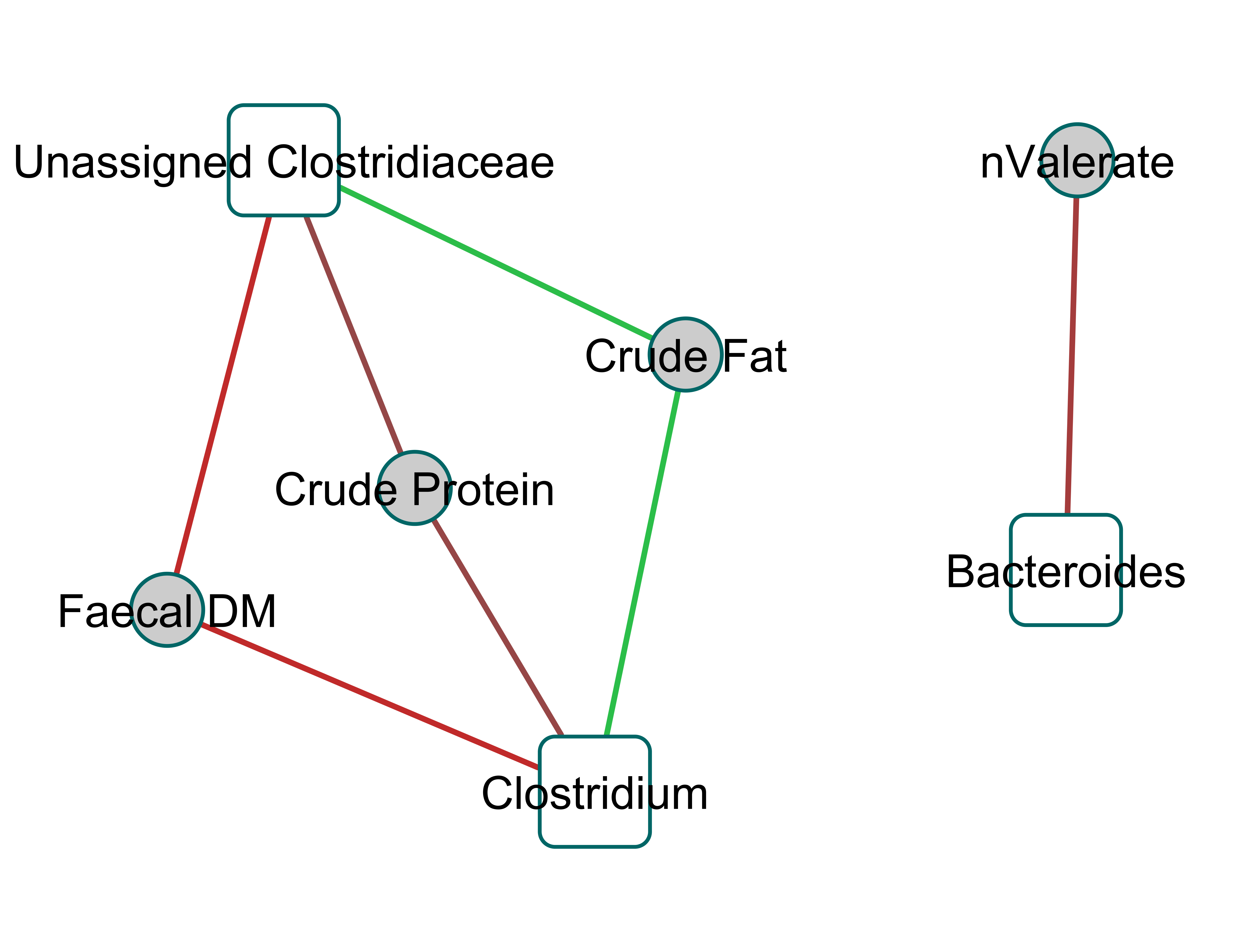

Supplement: Supplemental Information 6 — Includes physical measurement and metabolomics data (0.6 cutoff) revealing Clostridiaceae as a central node in dogs fed both the meat and kibbled diets. [file peerj-05-3019-s006.png]

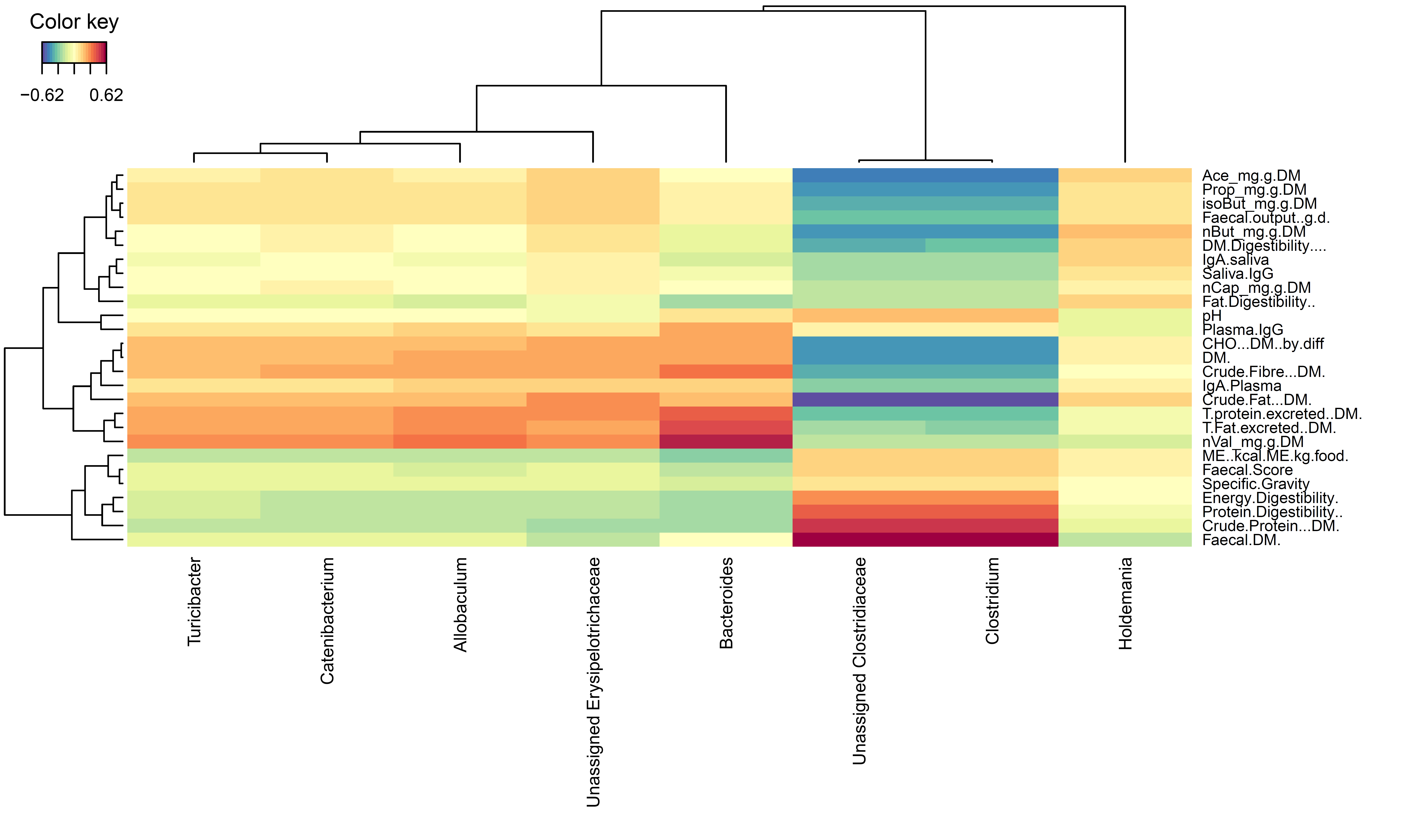

Supplement: Supplemental Information 7 — Correlation heat map describing the associations between Clostridiaceae (Clostridium and Unclassified Clostridiaceae), Erysipelotrichaceae (Allobaculum, Catenibacterium, Holdemania, Turicibacter and Unclassified Erysipelotrichaceae) and Bacteriodaceae (Bacteroides) levels in dogs and physiological markers of intestinal function fed both the kibbled and meat diet. Correlations greater than 0.50 were considered highly positively correlated, whereas correlations below −0.50 were considered to be highly negatively correlated. [file peerj-05-3019-s007.png]
